# Variable wildfire impacts on the seasonal water temperatures of western US streams: A retrospective study

**DOI:** 10.1371/journal.pone.0268452

**Published:** 2022-07-20

**Authors:** Mussie T. Beyene, Scott G. Leibowitz, Marcia Snyder, Joseph L. Ebersole, Vance W. Almquist

**Affiliations:** 1 Oak Ridge Institute for Science and Education (ORISE) Post-doc, c/o U.S. Environmental Protection Agency, Corvallis, Oregon, United States of America; 2 U.S. Environmental Protection Agency, Center for Public Health and Environmental Assessment, Corvallis, Oregon, United States of America; 3 U.S. Department of Agriculture Forest Service, Pacific Northwest Research Station, Corvallis, Oregon, United States of America; 4 Soil Health Institute, Corvallis, Oregon, United States of America; Sun Yat-Sen University, CHINA

## Abstract

Recent increases in the burn area and severity of wildfires in the western US have raised concerns about the impact on stream water temperature–a key determinant of cold-water fish habitats. However, the effect on seasonal water temperatures of concern, including winter and summer, are not fully understood. In this study, we assessed the impact of wildfire burns at Boulder Creek (Oregon), Elk Creek (Oregon), and Gibbon River (Wyoming) watersheds on the downstream winter and summer water temperatures for the first three post-fire years. To obtain results independent of the choice of the analytical method, we evaluated the consequence of each burn using three different statistical approaches that utilize local water temperature data. Our results from the three approaches indicated that the response of water temperatures to wildfire burns varied across seasons and sites. Wildfire burns were associated with a median increase of up to 0.56°C (Standard Error; S.E. < 0.23°C) in the summer mean water temperatures (MWT) and 62 degree-day Celsius (DDC; S.E. < 20.7 DDC) in the summer accumulated degree days (ADD) for the three subsequent years across studied stream sites. Interestingly, these burns also corresponded to a median decrease of up to 0.49°C (S.E. < 0.45°C) in the winter MWT and 39 DDC (S.E. < 40.5 DDC) in the winter ADD for the same period across sites. Wildfire effects on the downstream water temperatures diminished with increasing site distance from the burn perimeter. Our analyses demonstrated that analytical methods that utilize local watershed data could be applied to evaluate fire effects on downstream water temperatures.

## Introduction

In the western United States (WUS), salmonids are among the societally and ecologically important fishes that inhabit cold water. Life history forms of salmonids have evolved to optimize survival under local stream conditions, with water temperature being a primary constraint [1, 2]. Water resource managers in the WUS are concerned about potential landscape and climate change impacts on the thermal regime of streams as changes in winter and summer stream temperature outside of the salmonids’ optimum ranges can induce a host of challenges [3]. The warming of summer water temperatures can cause increased metabolic demands, reduced scope for growth, and lowered ability to resist disease in salmonids [4]. Increases in stream temperature can further affect salmonids by favoring warm-water species that prey upon or compete with them [5]. Very low water temperatures can also induce cessation of feeding and growth losses in salmonids [6].

Since the mid-1980s, large, high-severity wildfires have become more pervasive across the WUS due to the commingling effects of a warming climate, rapid wildland-urban interface expansion, and excessive forest fuel build-up [7]. High-severity riparian fires burn forest stands that shade streams, stabilize stream banks, and trap sediment and precipitation. This, in turn, modifies (at different scales) the heat inputs from processes (e.g., advective, radiative) that contribute to stream heat budgets and water temperature. Post-fire riparian vegetation losses typically lead to lower (riparian) shade and higher insolation for streams [2, 8, 9]. However, any effect of higher insolation can be attenuated by increases in stream heat loss due to post-fire increases in long-wave radiation and turbulent heat flux [10]. Post-fire vegetation losses and soil hydraulic property changes also enhance surface and subsurface flows into streams [11, 12], thereby reducing rates of stream heating [8, 13]. Lastly, overstory canopy burns have been related to increases in winter snow (with high albedo) accumulation on forest floors [14, 15], which decreases the amount of winter solar radiation reaching stream surfaces. The importance of radiative and advective heat inputs on the surface water temperature varies spatially and temporally due to local (e.g., shade, hydrology) and regional (e.g., solar radiation, climate) factors [16–18]; as such, thermal responses of streams to wildfire burns can be highly variable across seasons, stream reaches, and watersheds.

There have been many studies regarding the effect of high-severity riparian fires on summer water temperatures of small headwater streams [3, 8, 9, 19–28]. Their findings indicated that high-severity riparian burns were generally related to elevated summer water temperatures due to increased insolation. However, reported post-fire summer water temperature increases also range between 0.04–10°C across streams, and this variability across stream reaches and watersheds has been related to pyrologic, geomorphologic, hydrologic, and climatic factors. Dunham et al., [8] linked the disparity in the post-fire increase in the summer water temperatures of burned stream sites within the Boise River Basin in central Idaho (USA) to differences in the burn severity and fire-related channel morphology changes. Mahlum et al., [21] noted that in the Bitterroot Basin, Montana (USA), the stream site distance from the scorched riparian area influenced whether the post-fire summer water temperatures were warmer following wildfires in 2000. Koontz et al., [28] attributed the variations in the post-fire response of summer stream thermal regime across burned Pacific Northwest (PNW) watersheds to in situ streamflow and precipitation patterns.

Despite winter being a season during which water temperatures can strongly regulate physiological processes, including the development of fish embryos and subsequent emergence phenology [29], we are aware of only three publications [22, 23, 28] that have evaluated the post-fire response of winter water temperatures. Moreover, the findings of the studies conflicted. Rhoades et al. [22] did not detect any changes (relative to unburned sites) in the first-year average winter water temperatures of five stream reaches within the burned upper South Platte watershed in Colorado (USA) following the Hayman fires in 2002. Conversely, Koontz et al. [28] and Rhoades et al., [23] found that wildfires were associated with warmer winter water temperatures. Koontz et al. [28] also attributed the inter-site variability of the post-fire winter water temperature warmings to the extent of watershed burned.

Empirical studies often favor using paired watershed (control and treatment) comparisons, in a before-after-control-impact design, to assess disturbance effects on the water quality of unregulated (or reference, naturally flowing) streams [30]. The paired watershed comparisons approach allows estimation of the portion of stream temperature change related to the fire disturbance, simply as the change in the inter-watershed (burned and unburned) stream water temperature difference after a wildfire [8]. However, the paired watershed comparisons approach may have limited usability for evaluating the water temperature response in many burned WUS stream sites for two main reasons. The first reason is that most streams are ungauged and even those with high quality stream monitoring gages with pre-fire water temperature data are often found in watersheds where human influences on the flow and water temperature are substantial [31]. Another reason is that a stream site’s water temperature dynamics is influenced by in-situ conditions such as riparian density, channel width and orientation, hydrology, and groundwater contribution [13, 17], which can vary even among nearby stream reaches. This limitation necessitates the development of alternative analytical approaches for assessing fire effects on water temperatures in the western US.

Aside from the paired watershed comparison approach, we are not aware of any approach that has been advanced for detecting fire effects on the thermal regime of unregulated streams. Nevertheless, various statistical methods that utilize local stream data have been successfully developed or refined to detect, model, and predict the weather-related changes in water temperature under undisturbed conditions. Simple air-water temperature linear regression models have extensively been used to characterize the response of weekly or monthly water temperatures to air temperature changes [e.g., [32–34]]. Non-parametric models (e.g., k-nearest neighbor method) and machine learning models (e.g., random forest regression) conditioned on multiple weather variables have also been fitted to accurately predict stream water temperatures for periods ranging from hourly to annual means [e.g., [35–37]]. Given that the post-fire water temperature changes in unregulated streams are outcomes of both weather and fire influences, we can utilize these statistical approaches to analyze fire contributions. This study aims to demonstrate the applicability of some of these statistical approaches in evaluating the effect of wildfires on winter and summer water temperatures.

The objective of this study was to assess the effect of watershed burns on the downstream winter and summer water temperatures, with a focus on understanding the diversity in water temperature responses across seasons and sites. To this end, we utilized water temperature data from sites downstream of three burned western US watersheds. To get results independent of the choice of method, we employed three different analytical approaches —similar to the ones implemented by Beyene et al., [38] —to analyze the burn effect of each wildfire burn on the downstream water temperatures. The two key questions with regards to the main objective are: (a) How do wildfires influence winter and summer stream temperatures? In particular, do wildfires consistently result in warmer summer and winter stream temperatures? (b) Does the burn history (e.g., burn area, severity, distance from burn perimeter) and hydrology of a watershed influence the local stream temperature response?

## Methods

### Site selection and data source

**Fig 1:**
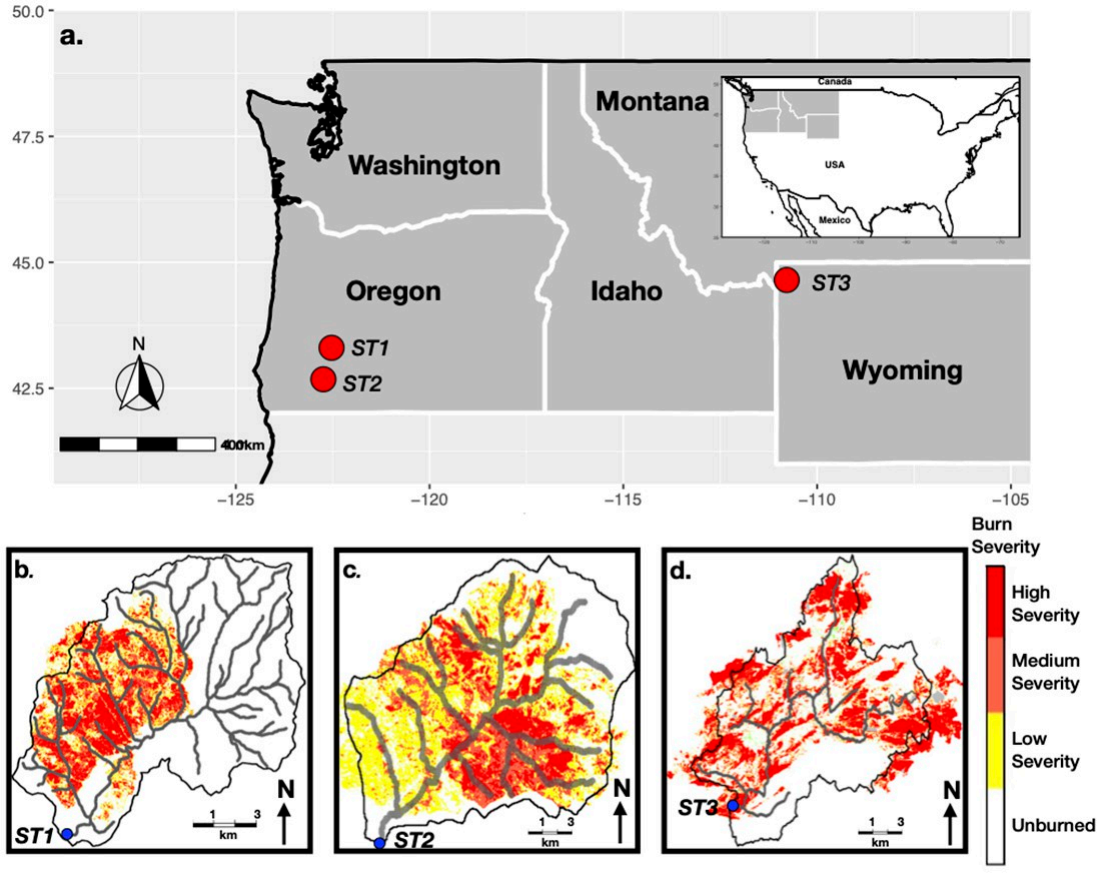
Location of studied stream sites and their burned watersheds. (a) Location of stream sites within the northwestern United States. The extent and severity of wildfire burn at (b) Elk Creek, (c) Boulder Creek, and (d) Gibbon River stream site watersheds. Blue filled circles represent the location of selected sites.

To assess wildfire impacts on downstream water temperatures, we first obtained geospatial wildfire data for the entire US, prepared and compiled by the Monitoring Trends for Burn Severity [MTBS; [39]] project. MTBS project databases currently have the most extensive compilation of burn extent and severity records for wildland fires of size greater than 4 km^2^ in the WUS from 1984 to 2018. Next, we cross-referenced the wildfire burn perimeters with the watershed delineations of US Geological Survey (USGS) Geospatial Attributes of Gages for Evaluating Streamflow version 2 [GAGES II; [40]] stream sites to determine burned USGS watersheds with water temperature data. Finally, we selected three WUS watersheds that each recorded extensive high-severity burns, did not contain any anthropogenic thermal or hydraulic control structures, and had at least seven years (Pre–fire ≥ 3 years, Post—fire = 3 years) of continuous daily water temperature and flow data Fig 1a–1d and Table 1.

**Table 1:** Physiographic, climatic, hydrologic, and burn characteristics of studied gages. The climatology was based on the 1980–2010 period. The daily mean discharge and monthly mean baseflow index represent the pre-fire streamflow condition.

USGS Gage Site	State	Study Years	Watershed Area (km^2^)	Fire Year	Percent Riparian Area Burned	Percent Riparian Area Burned Under High Severity	Site Distance Downstream From Burn Perimeter (m)	Average Monthly Air Temperature (°C)	Annual Precipitation Total (mm)	Average Daily Mean Streamflow (m^3^/sec)	Average Monthly Baseflow Index (%)
Boulder Creek (USGS-14316495)	Oregon	2001–2012	78	2008	81	31	138	8.6	1468	2.5	46
Gibbon River (USGS-06037500)	Wyoming	1984–1991	290	1988	83	22	0	0.7	665	3.3	81
Elk Creek (USGS-14338000)	Oregon	1997–2005	334	2002	32	13.1	2500	11	1206	6.2	41

We also accessed several national datasets to derive the burn history, hydrologic, climatic, and topographic data for selected watersheds. We extracted the spatial attributes of the wildfire burn area and severity for studied watersheds from MTBS project burn severity rasters. We computed the physiographic (Elevation, Slope) data for selected watersheds from the National Hydrography Dataset Plus-version 2 [NHDPlus V2; [41]] dataset. In addition, we estimated the watershed averaged daily mean air temperature and precipitation total for the 1979–2018 period from the gridded Parameter–elevation Regressions on Independent Slopes Model [PRISM; [42]] dataset. We also derived monthly accumulated potential evapotranspiration (PET) total for studied watersheds from the University of Idaho’s Gridded Surface Meteorological Dataset [GRIDMET; [43]]. We collected daily stream water flow and temperature data from USGS GAGES II stream sites within burned watersheds. Lastly, we obtained land cover data for our watersheds from the 2001 National Land Cover Database [NLCD; [44]] rasters. S1 Table summarizes the attributes of the datasets used in this study, including their sources.

### Site description

#### Boulder Creek near Tokettee Falls (USGS-14316495)

The Boulder Creek near Toketee Falls stream site is located 120 km east of Roseburg, in southwestern Oregon. Its watershed encompasses 78 km^2^ of land area, which, for the most part, lies within the western Cascade geologic province of southern Oregon. The watershed also spans in elevation from 500–1860 m above sea level (ASL) and has a mean slope of 43.9%. Nearly 92% of the watershed for Boulder Creek near Toketee Falls stream site lies within the federally protected Boulder Creek Wilderness [44]. Cultivated and developed land areas cover less than 0.01% of the watershed area [44].

According to the Koppen Geiger Climate Classification [45], the Boulder Creek watershed is found within the marine west coast climate zone, with mild climate due to the influence of Pacific Ocean winds. The watershed averaged mean air temperature ranges between 7–11°C annually, with monthly mean air temperatures as low as -1.3°C in January and as high as 20°C in August. The watershed averaged total annual precipitation is also about 1600 mm, with 62%–70% of that precipitation happening between November and March. While the watershed receives an average of 975 mm of total snowfall per year, most winter precipitation falls as rain. Summer rainstorms occur occasionally and are usually of short duration and limited areal coverage.

The annual stream hydrograph at the stream site is dominated by rain and snowmelt events. The high flow period begins about mid-November and generally lasts through April. Low flows usually prevail from July to September or October, which is a period of low precipitation. Flow at this stream site can be highly variable, ranging from as low as 0.1 m^3^/sec in August to 6.3 m^3^/sec in January.

#### Gibbon River near West Yellowstone (USGS- 06037000)

The Gibbon River near West Yellowstone stream site in northwest Wyoming (USA) has a watershed area of 290 km^2^. Its elevation ranges from 2116 to 2876 m ASL and has an average watershed slope of 13.2%. The watershed is entirely found within Yellowstone National Park, and forests and shrubs cover over 85% of the watershed [44].

The Gibbon River watershed lies within the continental subarctic climate zone with long, frigid winters and brief cool summers [45]. The watershed averaged mean annual air temperature ranges between 3–4°C with average monthly air temperatures as low as -10°C in January and as high as 17°C in August. The watershed averaged total yearly precipitation amount is about 600 mm, with average monthly precipitation as high as 73 mm in May and as low as 30 mm in August. Winter precipitation at the Gibbon River watershed is in the form of snow.

At the stream site, the spring snowmelt season coincides with the period of heavy rains, and thus peak streamflow occurs during late May and June. Summer storm events can also cause intermittent flow peaks, although the river flow usually starts to recede in late June and reaches a relatively constant level by the middle of July. Low flow conditions prevail from late summer until the beginning of spring snowmelt. Average baseflow contribution on the winter and summer streamflow ranges between 89–94%.

#### Elk Creek at Trail (USGS- 14338000)

The Elk Creek at Trail stream site is located 130 km south of Roseburg in southwestern Oregon. It has a watershed area of 334 km^2^. Almost all of the watershed lies in the western Cascade geologic province, which is dominated by the Tyee and Umpqua formation sandstones and siltstones that easily weather to clayey rich soil [46]. Elevations in the watershed range between 460–1765 m ASL with an average watershed gradient of 36.4%. Forests and shrubs cover 85% of the watershed land area, and a third of the watershed area is federally designated National Forest lands [44]. Cultivated and developed land areas cover less than 0.1% of the watershed area [44].

The Elk Creek watershed is located within the warm-summer Mediterranean climate with rainy winter and warm and dry summers [45]. Watershed averaged monthly mean air temperatures typically start the year as low as 0°C in January and reach their maximum of 20°C in July. Watershed averaged annual precipitation totals can range between 740–2000 mm, with over 66% of the yearly precipitation falling between November and March. Precipitation predominantly occurs as rain for elevations below 600 meters and rain/snow mix for elevations above 600 meters.

The streamflow regime at the stream site is similar to its precipitation pattern: streamflow is high in winter from rain and in spring from snowmelt, and low in the dry summer season. However, runoff generally lags behind precipitation by about a month. Baseflow contribution on daily streamflow ranges from 60% in the summer to 34% in winter.

### Stream water temperature indices

We summarized the daily time series for winter and summer water temperatures by calculating the winter and summer Mean Water Temperature (MWT) and the Accumulated Degree Days (ADD)—the cumulative sum of the difference between daily mean water temperature and 0°C. We computed winter and summer ADDs because cumulative water temperature measures have been used to predict the physiological development of coldwater fishes in streams [9]. In this study, the winter season refers to December through February, and the summer season represents July to September. The pre-fire period also referred to the 3–7 years preceding a fire event(s), while the post-fire period was used to denote the three immediate years after a fire.

### Analysis of the post-fire change in winter and summer stream water temperature indices

We adopted an analytical framework—a modification of the analytical framework implemented by Beyene et al., [38]—to assess wildfire burn effects on winter and summer water temperatures. This framework incorporated three different analytical approaches to evaluate whether watershed burning affected downstream water temperatures.

- Bootstrap method to analyze the post-fire changes in seasonal water and air temperature.
- Air-water temperature regression analysis to detect significant post-fire changes in weekly (e.g., October 1–7, 8–14) air-to-water temperature relations.
- Random forest regression model-based attribution approach to estimate the portion of the post-fire change in water temperature related to wildfire.

We employed these three analytical approaches for two main reasons. The first reason is that we wanted to obtain results that are independent of the choice of the analytical approach. The second reason is that we wanted to demonstrate the utility of analytical approaches that utilize local data to assess the post-fire change in stream water temperatures.

#### Bootstrap method

We used the bootstrap method [47] to determine the post-fire change in the seasonal (winter and summer) water temperature (MWT and ADD) or weather (precipitation and air temperature) index for each site. The advantage of applying this procedure is that it did not require making any assumptions or estimating any parameter when simulating the pre-fire range for seasonal weather or water temperature index. Instead, we repeatedly shuffled (with replacement) the pre-fire years to generate 1000 subsamples with three pre-fire years. Next, we determined the seasonal weather or water temperature index for each pre-fire year and the median seasonal index for each subsample. Lastly, we estimated the post-fire change in the seasonal weather or water temperature index as the difference between the median of post-fire seasonal weather or water temperature index (*n* = 3) and the 500^th^ ranked (or median) sample value. We also assumed the post-fire seasonal weather or water temperature index to be significantly changed at the 0.05^th^ significance level if the median of the post-fire seasonal weather or water temperature index was either less than the 25^th^ or greater than the 975^th^ ranked subsample value.

#### Air-water temperature regression analysis

We developed and compared the weekly mean air temperature-water temperature (e.g., MWT, ADD) regression lines for the pre- and post-fire periods to characterize wildfire effects on the sensitivity of weekly winter and summer stream water indices to air temperature changes. To this end, we first fitted two dynamic regression lines [48] that described the weekly winter (or summer) mean air temperature-water temperature relations for the pre- and post-fire years separately (restricted) and collectively (unrestricted). These regression lines were of the form

$$\begin{array}{c} T_{water}=\beta_{0}+\beta_{1}*\left(1-X\right)+\beta_{2}*\left(1-X\right)*T_{air}+\beta_{3}*X*T_{air}+\epsilon , \end{array}$$ (1)

where *β* represents parameter estimates (coefficients), and T_*air*_ and T_*water*_ denote weekly mean air temperature (MAT) and water temperature, respectively. *X* is a time parameter that equals one in the unrestricted regression line. If the observation year precedes or follows the fire year in the restricted regression line, *X* equals zero and one, respectively. *ϵ* is the residual term of the regression line that is modeled by the seasonal Auto-Regressive Integrated Moving Average model [SARIMA; [49]]. The SARIMA model function is described by (*p*, *d*, *q*)(*P*, *D*, *Q*)_*n*_ where (*p*, *d*, *q*) is the non-seasonal component of the model and (*P*, *D*, *Q*) is the seasonal component of the model. Moreover, *p* represents the order of non-seasonal autoregression, *d* signifies the order of non-seasonal differences needed for non-stationarity, *q* denotes the order of non-seasonal moving average, *P* represents the order of seasonal autoregression, and *D* signifies the order of seasonal differencing, and *Q* denote the order of seasonal moving average. *n* is the length of the season, and *n* equals 13 in this study given that there were 13 weeks in a winter or summer season. The *forecast* package [50] in the *R* computing environment provided the optimizing algorithm to estimate the coefficients in these regression lines. We also evaluated the compliance of our models for normality of residuals using the Shapiro-Wilk normality test, outliers with the Bonferroni test, and homogeneity of variance using the non-constant variance score test.

We conducted Likelihood Ratio Tests [LRT; [51]] to compare the relative goodness of fit of the restricted and unrestricted regression lines for the inter-weekly water temperature variability. In this test, the null hypothesis was that riparian burns did not affect the air-water temperature relations, and thus the restricted regression line would not offer an appreciable improvement over the unrestricted regression line in representing the pre- and post-fire weekly stream temperature variability for the studied site. We rejected the null hypothesis in this study if the LRT score fell below 0.05.

#### Random forest regression model-based attribution approach

Random forest [52] is a machine learning algorithm capable of handling large nonlinear, noisy, fragmented, or correlated multidimensional data for classification or regression. A random forest regression (RFR) model is built by constructing ensembles of regression trees trained using recursive subsets of all observations, and predictions are computed as the expected value of all individual predictions from each tree in the random forest model [53]. In this study, we developed RFR models for each stream site (*n* = 3) and season (*n* = 2), a total of six RFR models, to estimate the portion of the post-fire change in seasonal (here winter, summer) MWT and ADD attributed to fire effects. This approach involved three steps. For each stream site and season, we first constructed multiple RFR models for daily MWT using daily-to-seasonal weather (precipitation, potential evapotranspiration, and air temperature) variables and daily stream water temperature data for the pre-fire period. We then selected the best fit RFR model for winter or summer MWT (or ADD) based on their relative fitness for each site. We evaluated the performances of these models in predicting the winter or summer MWT (or ADD) for each site under undisturbed (or pre-fire conditions) using the Leave One Out Cross Validation (LOOCV) experiments. Next, we input weather data into the best fit RFR models for each site and season to predict the winter or summer MWT (or ADD) for each post-fire year. Lastly, we computed the fire-related change in the seasonal MWT (or ADD) for each site as the median difference between observed and predicted seasonal MWT (or ADD) for the three post-fire years.

During RFR model building for daily winter or summer MWTs, we considered daily-to-seasonal weather (air temperature, precipitation, potential evapotranspiration) variables as possible covariates. Many studies have demonstrated that the daily air temperature serves as a good proxy of the atmospheric heat input into streams and thus can be a good predictor of the daily water temperature [e.g., [32, 54]]. Runoff and groundwater input can also have a notable effect on the daily winter and summer water temperatures of studied streams as streamflow at these sites generally are runoff-dominated in the winter and groundwater-dominated in the summer. Hence, we added a 1–200 day rolling average (ending on the given day) of daily precipitation and potential evapotranspiration totals as possible explanatory variables. We also included a 30–200 day rolling average (ending on the given day) of daily mean air temperature as potential predictors since prior studies [e.g., [35]] have demonstrated that these could be used to infer shallow groundwater influences and hyporheic exchanges on stream water temperatures. We fitted multiple sets of candidate RFR models using one or more of these predictor variables. The maximum number of covariates included in any candidate model for daily MWT was set at three to avoid overfitting. *Pearson*’s correlation between any covariates within any candidate model was also set not to exceed 0.7 to eliminate multicollinearity effects. The *randomForest* implementation [55] in the *R* computing environment provided the optimizing algorithm to develop RFR models for daily MWTs and ADDs for each site.

We used the bias-corrected Akaike Information Criterion [AICc; [56]] to compare the complexity and goodness of fit of candidate RFR models for each site and season. Best fit RFR models were selected as those with the least AICc score. We then assessed the predictive performance (skills) of the best fit RFR models under undisturbed scenarios using the LOOCV approach. This approach involved withholding the weather and water temperature data for each pre-fire year, re-calibrating the RFR model to the remaining data and making the winter or summer MWT (and ADD) predictions for the left-out year, and then repeating the procedure for all pre-fire years. We compared LOOCV predicted winter and summer MWT and ADD values with that of the observed using three model performance metrics: Nash-Sutcliffe coefficient of model efficiency [NSC; [57]], bias, and root mean squared error (RMSE). NSE measures the total residual error relative to the total variance within the data. Bias estimates the tendency of a model to over predict (bias < 0) and underpredict (bias > 0). RMSE measures the absolute error associated with each model.

## Results

### Bootstrap approach

The post-fire changes in the winter MWT and ADD were contrasting across the three studied stream sites. For the three post-fire years, winter MWTs were lower on average by 0.62°C for the Boulder Creek stream site and 0.15°C for the Gibbon River site than the pre-fire (Table 2). Unsurprisingly, the post-fire winter ADD was also lower than that of the pre-fire for both sites. In contrast, post-fire winter MWTs and ADDs for the Elk Creek site were higher on average by 0.92°C and 95.7 DDC, respectively, than the pre-fire period (Table 2). The post-fire changes in the winter MWT and ADD were statistically (*p*< 0.05) significant only for the Boulder Creek and Elk Creek stream sites (Table 2).

**Table 2:** Median differences between the pre- and post-fire winter (December-February; upper lines) and summer (July-September; bottom lines) weather, water temperature, and flow conditions. **Bold** text represents statistically significant change (*p*< 0.05).

USGS Gage Site	Mean Water Temperature (°C)	Accumulated Degree-days (DDC)	Air Temperature (°C)	Precipitation Total (mm)	Streamflow (m^3^/sec)	Baseflow Index (%)
Boulder Creek (USGS- 14316495)	-0.6	**-73**	-0.2	-119	-0.4	0.5
	**0.5**	**46**	-0.1	-21	**0.13**	-1.2
Gibbon River (USGS-06037000)	-0.2	-24	1.1	-2.7	**-0.3**	-0.7
	**1.5**	**136**	**2.4**	**-56**	-0.3	**1.6**
Elk Creek (USGS-14338000)	**0.9**	**96**	**1.7**	-55	-2.5	-3.2
	**0.6**	**60**	1.0	0.7	0	**10**

The post-fire summer MWTs and ADDs were significantly (*p*< 0.05) higher than the pre-fire for the three stream sites (Table 2). For the three post-fire years, the average increase in the summer MWTs ranged between 0.50–1.47°C, and the average increase in the summer ADDs ranged between 45–136 DDC across the three sites. We found the greatest post-fire increase in the summer MWT and ADD across the three stream sites at the Gibbon River site (Table 2).

**Fig 2:**
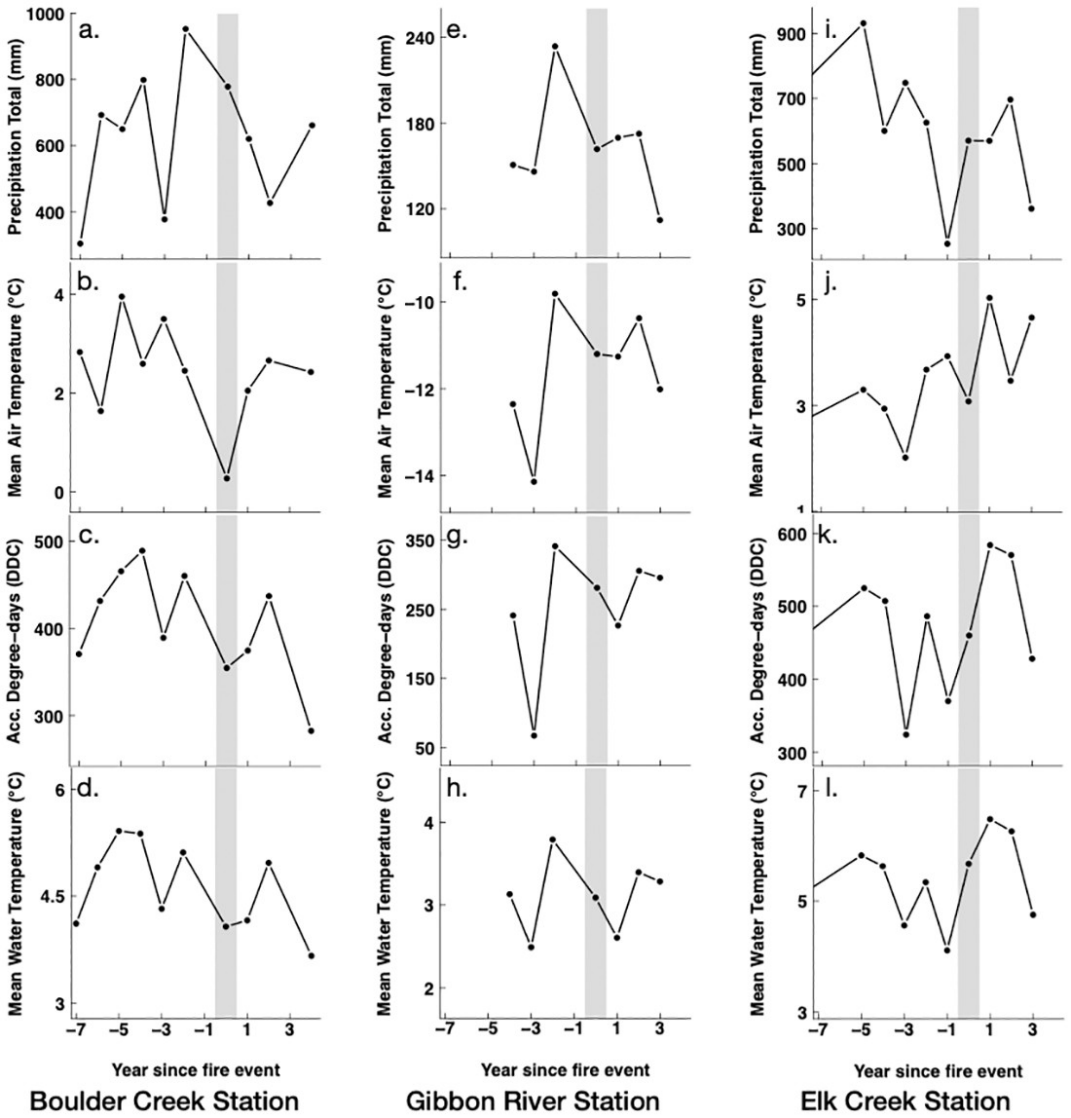
Times series of pre- and post-fire winter precipitation totals, mean air temperatures, accumulated degree days, and mean water temperatures at (a-d) Boulder Creek, (e-h) Gibbon River, and (i-l) Elk Creek sites. Grey line represents the fire year.

**Fig 3:**
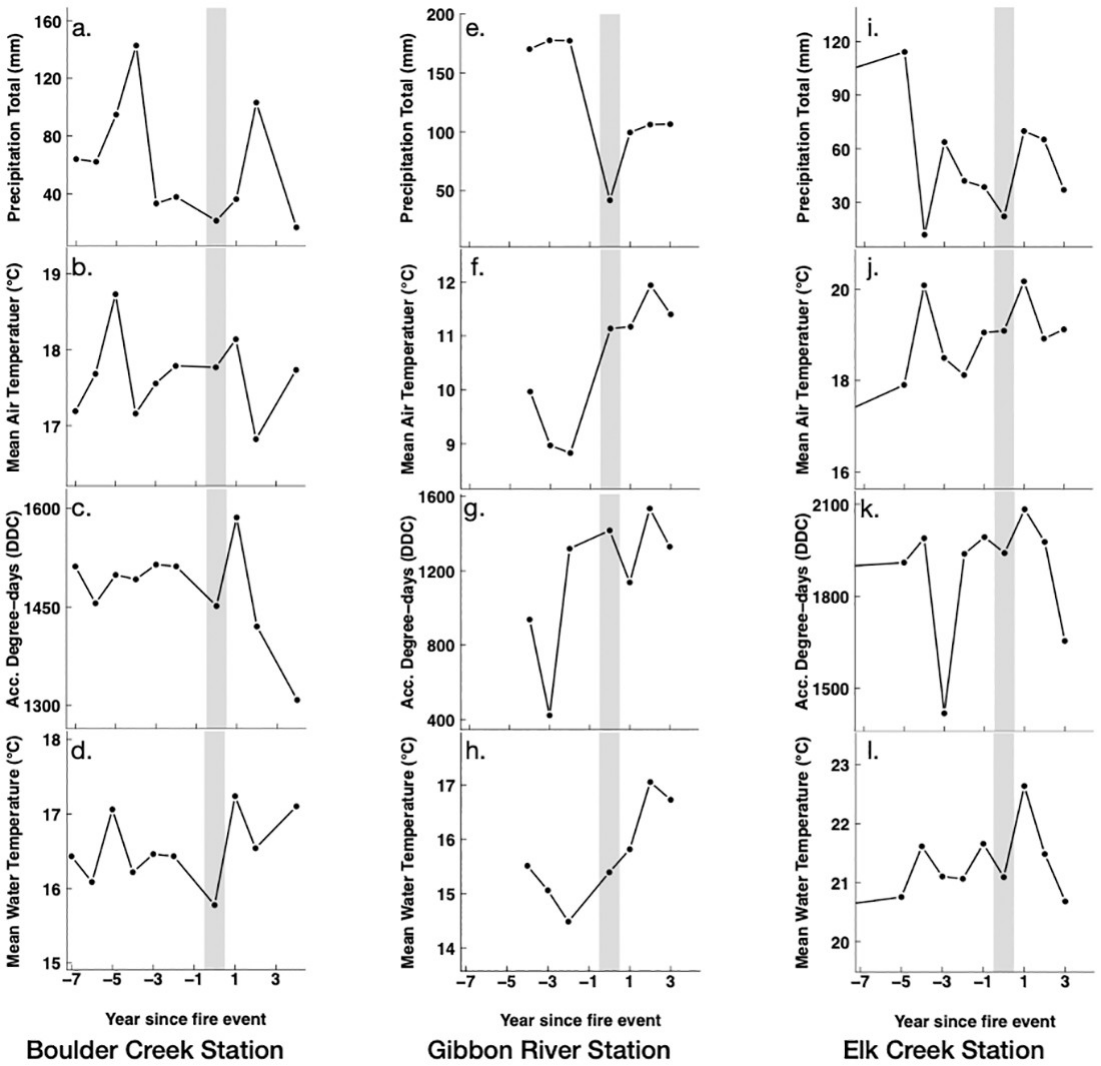
Times series of pre- and post-fire summer precipitation totals, mean air temperatures, accumulated degree days, and mean water temperatures at (a-d) Boulder Creek, (e-h) Gibbon River, and (i-l) Elk Creek sites. Grey line represents the fire year.

The post-fire changes in the winter and summer MWT and ADD across the three stream sites showed some correspondence to the post-fire changes in the summer air temperature and precipitation totals (Figs 2 and 3a–3l). All three streams with post-fire increases in summer MWTs and ADDs also recorded post-fire increases in the summer mean air temperatures (MATs) and post-fire decreases in the summer precipitation totals (Fig 3a–3l). Similarly, post-fire winter MATs were significantly (*p*< 0.05) higher for the Elk Creek site, which also showed an increase in its post-fire winter MWT and ADD (Fig 2c, 2f and 2i).

### Air-water temperature regression analysis

**Fig 4:**
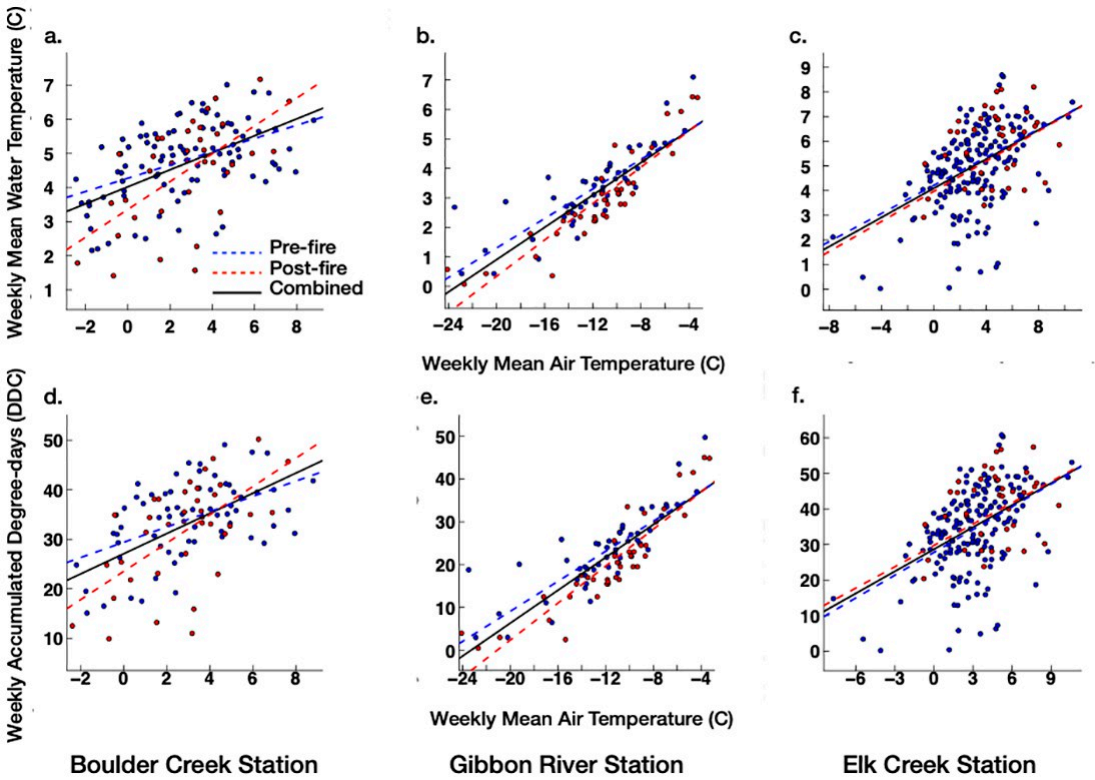
Pre- and post-fire regression lines for weekly winter mean air temperature (MAT)—mean water temperature (MWT) and accumulated degree-days (ADD) data for (a and d) Boulder Creek, (b and e) Gibbon River, and (c and f) Elk Creek stream sites. Red and blue filled circles represent the pre- and post-fire weekly winter MAT–MWT or ADD events, respectively.

Across the three studied streams, the unrestricted regression lines for pre-and post-fire weekly winter stream MWTs and ADDs represented 42–61% of their total variability, while the restricted regression lines accounted for 43–65% of their total variability (Tables 3 and 4). The LRT-based comparison of the two types of regression lines for the three sites indicated that the weekly winter MAT–MWT and ADD relations were significantly (*p*< 0.05) shifted following wildfire burns for the Gibbon River and Boulder Creek stream sites (Tables 3 and 4). Moreover, post-fire weekly winter MATs corresponded to lower weekly MWTs and ADDs than pre-fire over most of the weekly winter MAT ranges for these sites (Fig 4a–4f). In contrast, we did not detect a significant (*p*< 0.05) post-fire change in the weekly winter MAT-MWT or MAT-ADD relations for the Elk Creek stream site (Tables 3 and 4, Fig 4a–4f).

**Table 3:** Coefficients and fitness of restricted and unrestricted regression models for pre- and post-fire weekly winter air temperature—water temperature relations for studied stream sites. Sites in **bold** are those where the restricted regression model was significantly (*p*< 0.05) different from the unrestricted regression model.

USGS Gage Site	Unrestricted Model			Restricted Model					SARIMA Model for Residuals	Likelihood Ratio Test Score
	T_*air*_	*p*	R^2^	${\text{T}}_{air}^{pre}$	*p*	${\text{T}}_{air}^{post}$	*p*	R^2^		
**Boulder** **Creek** **(USGS-14316495)**	0.28	< 0.01	54	0.23	< 0.01	0.39	< 0.01	57	(1,0,0) (0,0,0)_13_	0.04
**Gibbon** **River** **(USGS-06037000)**	0.27	< 0.01	61	0.25	< 0.01	0.30	< 0.01	65	(1,0,0) (0,0,1)_13_	0.03
Elk Creek (USGS-14338000)	0.16	< 0.01	42	0.12	0.03	0.23	< 0.01	43	(1,0,1) (0,0,0)_13_	0.35

T_*air*_ stands for air temperature, ${\text{T}}_{air}^{pre}$ represents pre-fire air temperature, and ${\text{T}}_{air}^{post}$ signifies post-fire air temperature.

**Table 4:** Coefficients and fitness of restricted and unrestricted regression models for pre- and post-fire weekly winter air temperature—accumulated degree-days relations for studied stream sites. Sites in **bold** are those where the restricted regression model was significantly (*p*< 0.05) different from the unrestricted regression model.

USGS Gage Site	Unrestricted Model			Restricted Model					SARIMA Model for Residuals	Likelihood Ratio Test Score
	T_*air*_	*p*	R^2^	${\text{T}}_{air}^{pre}$	*p*	${\text{T}}_{air}^{post}$	*p*	R^2^		
**Boulder** **Creek** **(USGS-14316495)**	1.99	< 0.01	54	1.61	< 0.01	2.72	< 0.01	56	(1,0,0) (0,0,0)_13_	0.04
**Gibbon** **River** **(USGS-06037000)**	1.92	< 0.01	61	1.77	< 0.01	2.09	< 0.01	65	(1,0,0) (0,0,1)_13_	0.03
Elk Creek (USGS-14338000)	1.11	< 0.01	42	0.85	0.03	1.57	< 0.01	43	(1,0,1) (0,0,0)_13_	0.35

T_*air*_ stands for air temperature, ${\text{T}}_{air}^{pre}$ represents pre-fire air temperature, and ${\text{T}}_{air}^{post}$ signifies post-fire air temperature.

**Fig 5:**
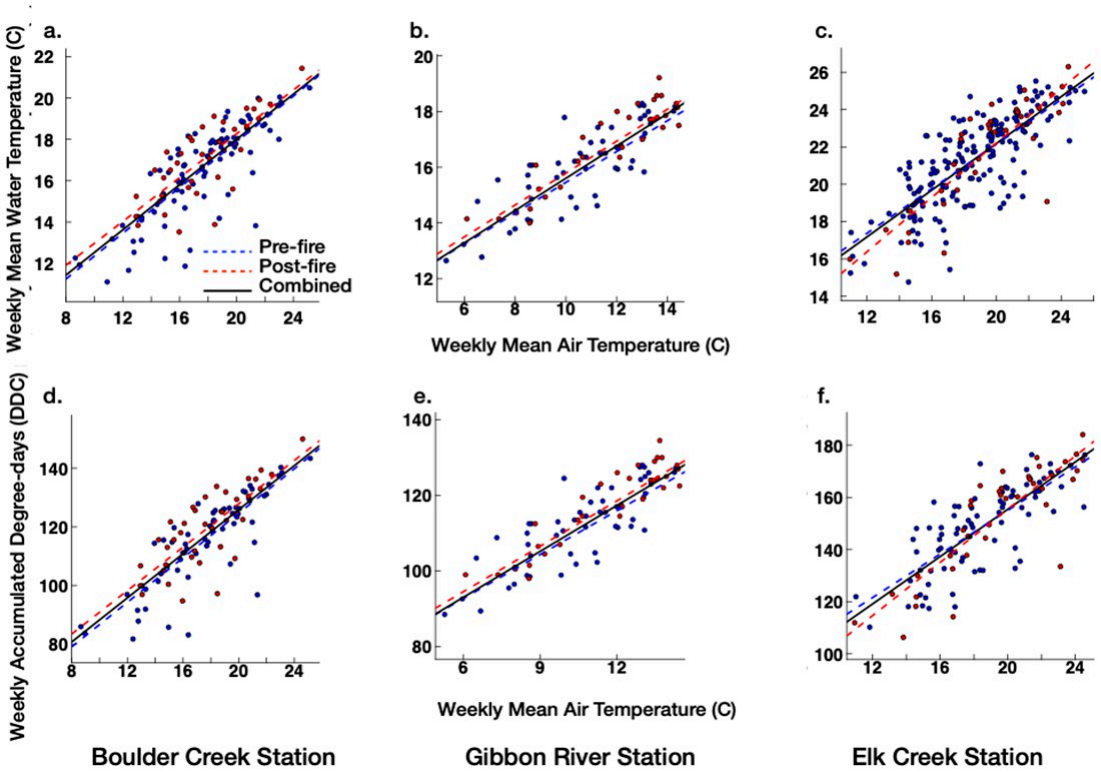
Pre- and post-fire regression lines for weekly summer mean air temperature(MAT)—mean water temperature (MWT) and accumulated degree days (ADD) data for (a and d) Boulder Creek, (b and e) Gibbon River, and (c and f) Elk Creek stream sites. Red and blue filled circles represent the pre- and post-fire weekly summer MAT–MWT or ADD events, respectively.

Across the three studied sites, the unrestricted regression lines for weekly summer MWTs and ADDs accounted for 67–85% of the total variability, while the restricted regression lines accounted for 68–87% of the total variability (Tables 5 and 6). The LRT-based comparison of these regression lines for each stream indicated significant changes in the post-fire weekly summer MAT-MWT and MAT-ADD relations for Boulder Creek and Gibbon River sites. Moreover, post-fire weekly summer MATs corresponded to higher weekly MWTs and ADDs than the pre-fire for the two stream sites (Fig 5a–5f). In contrast, we did not find a significant (*p*< 0.05) post-fire change in the weekly summer MAT-MWT or MAT-ADD relations for the Elk Creek site (Tables 5 and 6, Fig 5a–5f).

**Table 5:** Coefficients and fitness of restricted and unrestricted regression models for pre- and post-fire weekly summer air temperature—water temperature relations for studied stream sites. Sites in **bold** are those where the restricted regression model was significantly (*p*< 0.05) different from the unrestricted regression model.

USGS Gage Site	Unrestricted Model			Restricted Model					SARIMA Model for Residuals	Likelihood Ratio Test Score
	T_*air*_	*p*	R^2^	${\text{T}}_{air}^{pre}$	*p*	${\text{T}}_{air}^{post}$	*p*	R^2^		
**Boulder** **Creek** **(USGS-14316495)**	0.32	< 0.01	85	0.31	< 0.01	0.33	< 0.01	87	(1,0,0) (0,1,1)_13_	0.01
**Gibbon** **River** **(USGS-06037000)**	0.33	< 0.01	76	0.1	< 0.01	0.44	< 0.01	78	(1,1,0) (1,0,0)_13_	0.05
Elk Creek (USGS-14338000)	0.31	< 0.01	67	0.25	< 0.01	0.43	< 0.01	68	(0,0,1) (2,0,0)_13_	0.20

T_*air*_ stands for air temperature, ${\text{T}}_{air}^{pre}$ represents pre-fire air temperature, and ${\text{T}}_{air}^{post}$ signifies post-fire air temperature.

**Table 6:** Coefficients and fitness of restricted and unrestricted regression models for pre- and post-fire weekly winter air temperature—accumulated degree-days relations for studied stream sites. Sites in **bold** are those where the restricted regression model was significantly (*p*< 0.05) different from the unrestricted regression model.

USGS Gage Site	Unrestricted Model			Restricted Model					SARIMA Model for Residuals	Likelihood Ratio Test Score
	T_*air*_	*p*	R^2^	${\text{T}}_{air}^{pre}$	*p*	${\text{T}}_{air}^{post}$	*p*	R^2^		
**Boulder** **Creek** **(USGS-14316495)**	2.26	< 0.01	85	2.26	< 0.01	2.33	< 0.01	87	(1,0,0) (0,1,1)_13_	0.01
**Gibbon** **River** **(USGS-06037000)**	2.29	< 0.01	76	1.16	< 0.01	3.14	< 0.01	78	(1,1,0) (1,0,0)_13_	0.05
Elk Creek (USGS-14338000)	2.14	< 0.01	67	1.73	< 0.01	3.04	< 0.01	68	(0,0,1) (2,0,0)_13_	0.20

T_*air*_ stands for air temperature, ${\text{T}}_{air}^{pre}$ represents pre-fire air temperature, and ${\text{T}}_{air}^{post}$ signifies post-fire air temperature.

### Random forest regression model-based attribution approach

The best fit RFR models described the pre-fire weekly winter and summer MWT and ADD in training sets well and accounted for 88–100% of the total variability across the three streams (Tables 7 and 8). Moreover, the predictive skills for the best fit RFR model for each season and site during LOOCV evaluations were characterized by low RMSE and bias values and high NSCs (> 65%; Tables 7 and 8).

**Table 7:** Fitness, structure, and performance of best fit random forest regression models for the winter mean water temperatures and accumulated degree-days of studied stream sites.

USGS Gage Site	Random Forest Regression Model						
	Model Covariates	mtry	ntree	Correlation between Observed & Predicted	LOOCV Model Performance		
					RMSE (°C)	NSC (%)	Bias (°C)
Boulder Creek (USGS-14316495)	Daily mean air temperatures	2	318	1.0	0.11	95	-0.02
	7-day moving average air temperatures						
	October-March precipitation totals						
	January-June precipitation totals						
Gibbon River (USGS-06037000)	Daily mean air temperatures	1	258	1.0	0.12	95	0.12
	Daily potential evapo-transpiration totals						
Elk Creek (USGS-14338000)	Daily mean air temperatures	1	395	0.88	0.45	65	0.01
	October-March precipitation totals						

The abbreviation *LOOCV* stands for leave one out cross validation experiment. *mtry* also represents the number of predictor variable(s) randomly sampled for each tree in the RFR model, and *ntree* signifies the number of trees to grow in the RFR model.

**Table 8:** Fitness, structure, and performance of best fit random forest regression models for the winter mean water temperatures and accumulated degree-days of studied stream sites.

USGS Gage Site	Random Forest Regression Model						
	Model Covariates	mtry	ntree	Correlation between Observed & Predicted	LOOCV Model Performance		
					RMSE (°C)	NSC (%)	Bias (°C)
Boulder Creek (USGS-14316495)	Day of the year (e.g., October 1^st^ = 1)	5	500	0.93	0.17	74	-0.01
	Daily mean air temperatures						
	30-day moving average air temperatures						
Gibbon River (USGS-06037000)	Daily mean air temperatures	1	460	1	0.13	94	0.04
	30-day moving average air temperatures						
	180-day moving average air temperatures						
	150-day moving precipitation totals						
Elk Creek Site (USGS-14338000)	Day of the year	2	478	0.95	0.23	75	0.04
	Daily mean air temperatures						
	30-day moving average air temperatures						

The abbreviation *LOOCV* stands for leave one out cross validation experiment. *mtry* also represents the number of predictor variable(s) randomly sampled for each tree in the RFR model, and *ntree* signifies the number of trees to grow in the RFR model.

**Fig 6:**
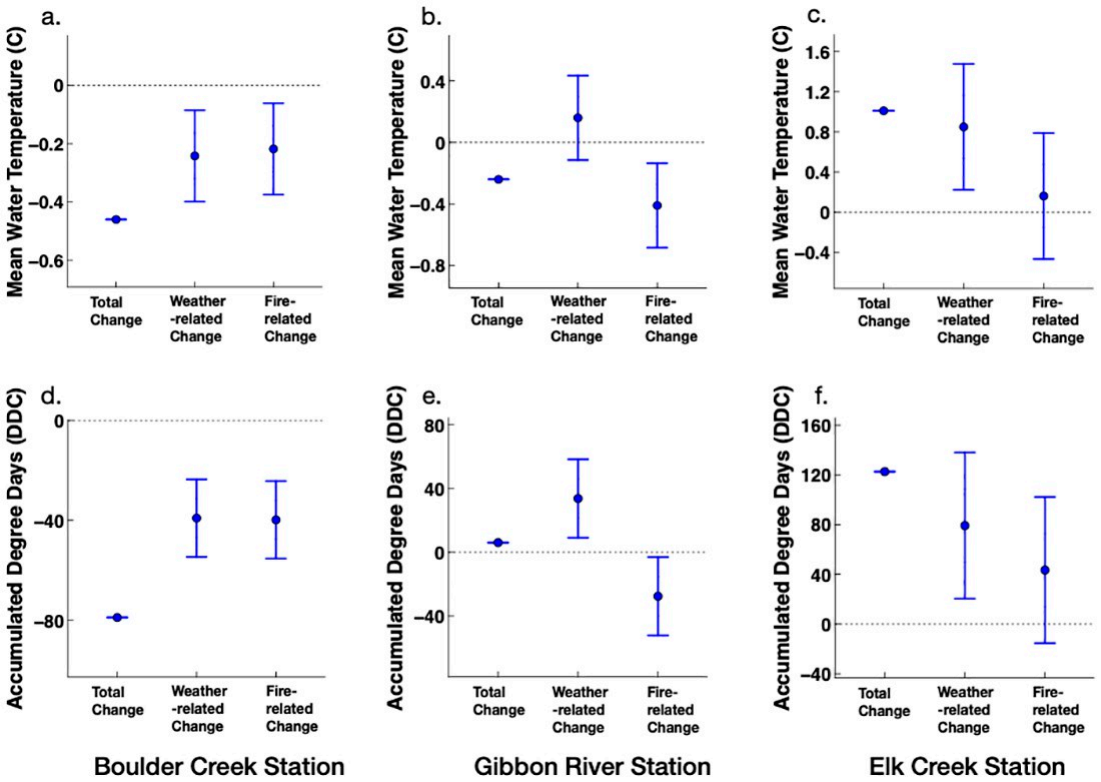
Weather and wildfire contributions on the post-fire change in the winter mean water temperature and accumulated degree days at (a, d) Boulder Creek, (b, e) Gibbon River, and (c, f) Elk Creek stream sites. Filled circles represent point estimates and the bars signify the 95% confidence intervals (1.96 x S.E.).

These models were then used to parse wildfire and weather-variability contributions on the post-fire winter and summer MWT and ADD changes. Results indicated that wildfire effects on the MWTs and ADDs varied based on the season and stream site. Wildfires were related to a median decrease in the winter MWT by 0.22°C (Standard Error = *S*.*E*. = 0.11°C) for the Boulder Creek stream site and 0.49 ºC (*S*.*E*. = 0.12°C) for the Gibbon River stream site for the three following years (Fig 6a, 6b, 6d and 6f). These fires were also associated with the lowering of the winter ADDs by 39.8 DDC (*S*.*E*. = 9.9 DDC) for the Boulder Creek site and 27 DDC (*S*.*E*. = 10.8 DDC) for the Gibbon River site for the three following years (Fig 6c and 6f). In contrast, the 2001 wildfire within the Elk Creek watershed corresponded to a median increase in the winter MWT by 0.16°C (*S*.*E*. = 0.45°C) and winter ADD by 43.5 DDC (*S*.*E*. = 40 DDC) for the three post-fire years for the studied downstream site (Fig 6a–6d).

**Fig 7:**
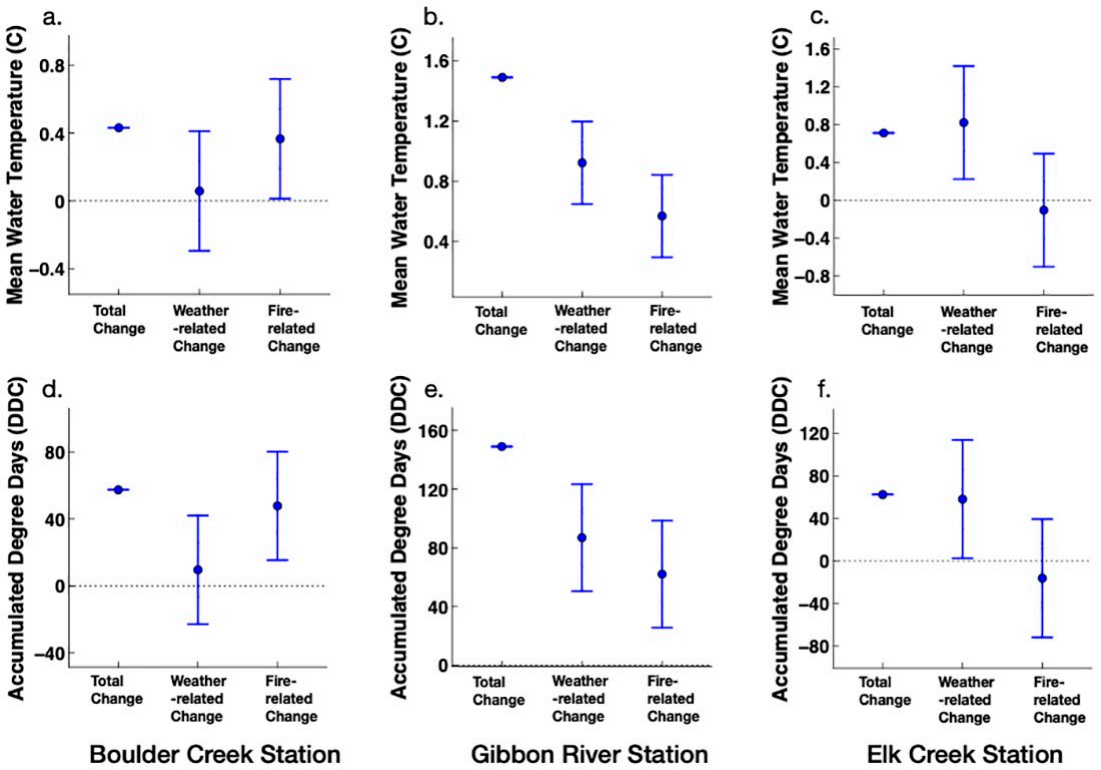
Weather and wildfire contributions on the post-fire change in the summer mean water temperature and accumulated degree days at (a, d) Boulder Creek, (b, e) Gibbon River, and (c, f) Elk Creek stream sites. Filled circles represent point estimates and the bars signify the 95% confidence intervals (1.96 x S.E.).

Wildfires were related to a median increase in the summer MWTs by 0.39°C (*S*.*E*. = 0.17°C) for the Boulder Creek site and 0.56°C (*S*.*E*. = 0.13°C) for the Gibbon River site for the three post-fire years (Fig 7a and 7b). These fires were also associated with elevating summer ADDs by 47.6 DDC (*S*.*E*. = 15.3 DDC) for the Boulder Creek site and 62 DDC (*S*.*E*. = 11.7 DDC) for the Gibbon River site (Fig 7d and 7e). On the other hand, wildfire burns at Elk Creek watershed corresponded to a median decrease in the summer MWT by 0.08°C (*S*.*E*. = 0.23°C) and a median decrease in the summer ADD by 16.3 DDC (*S*.*E*. = 20.7 DDC) for the three post-fire years for the studied downstream site (Fig 7c and 7f).

The difference in the pre- and post-fire weather conditions contributed to the post-fire changes in the winter and summer MWT and ADD of studied streams. Weather variability was related to a median decline in the winter MWT and ADD by 0.24°C (*S*.*E*. = 0.11°C) and 39.1 DDC (*S*.*E*. = 9.9 DDC), respectively, for the Boulder Creek site (Fig 6a and 6d). In contrast, post-fire weather conditions were associated with elevating winter MWTs and ADDs for Gibbon River and Elk Creek sites (Fig 6b, 6c, 6e and 6f). In addition, weather variability was related to a median increase in the summer MWT by 0.08°C (*S*.*E*. = 0.17°C) for Boulder Creek, 0.93°C (*S*.*E*. = 0.13°C) for Gibbon River, and 0.78°C (*S*.*E*. = 0.23°C) for Elk Creek stream sites for the three post-fire years (Fig 7a– 7c). The median weather-related increase in the summer ADDs for the three post-fire years ranged between 9.6–87 (*S*.*E*.< 20.7 DDC) across the three studied stream sites (Fig 7d–7f).

## Discussion

### Stream thermal response to wildfire effects

The results of our three separate analyses indicated that high-severity riparian burns affected the winter and summer water temperatures of stream sites within the Gibbon River (Wyoming) and Boulder Creek (Oregon) watersheds. Although the magnitudes of fire-related winter and summer water temperature change for the two stream sites were modest, this is to our knowledge the first study that has detected wildfire signals on the thermal regime of streams with a watershed area exceeding 135 km^2^. In addition, the results here imply that riparian burns could have contrasting effects on the winter and summer stream water temperatures, even after accounting for weather effects.

#### Winter season

Our analyses indicated that riparian fires within the Gibbon River (Wyoming) and Boulder Creek (Oregon) watersheds corresponded to a decline in winter MWTs and ADDs for studied downstream sites. In the case of the Gibbon River stream site, this was despite the average post-fire winter air temperature being slightly warmer (0.49°C) than the pre-fire (Table 2). Our RFR model-based results indicated that for the three post-fire years, the median fire-related decrease in winter MWT ranged between 0.22–0.49°C (*S*.*E*.< 0.12°C) and the median fire-related decrease in winter ADD ranged between 27–39.8 DDC (*S*.*E*.< 10.8 DDC), across the two sites.

There are several possible mechanisms by which high-severity riparian burn may cool winter water temperatures for the Boulder Creek and Gibbon River stream sites. The combustion of riparian shade could have advanced the winter heat loss from the two stream sites by increasing longwave radiation and turbulent fluxes [10]. The burning of overstory canopy promotes the accumulation of winter snowfall on forest floors [15, 58], which could have a lessening effect on the winter solar radiation reaching stream surfaces. In the case of the Boulder Creek stream site, winter precipitation also causes higher streamflow volumes, and greater connectivity with the unburnt stream reaches. This could have lowered the efficacy of higher winter insolation in influencing local winter water temperatures.

Our analyses also indicated that the 2002 riparian fires within the Elk Creek watershed did not have a substantial effect on the winter MWTs and ADDs for the studied downstream stream site. The absence of a significant fire-related change in winter MWTs and ADDs for this site could be attributed to many factors. The stream site is characterized by higher winter streamflow and steeper watershed slopes, which might reduce the efficacy of atmospheric (e.g., short and longwave radiation) heat fluxes in influencing winter water temperatures. The stream site was also 2.5 km downstream of the burned riparian area. This might have allowed groundwater inflow and riparian shade downstream of burned areas to moderate winter water temperature changes as the water flows from burned areas to unburned areas. In addition, factors such as differences in the watershed area and riparian and topographic shade are likely to have contributed to the smaller wildfire effect on the winter MWT and ADD for this site.

Our analyses indicated that post-fire weather patterns had contrasting influences on the fire-related changes in winter stream temperatures across studied stream sites. In the case of Boulder Creek and Elk Creek sites, weather conditions enhanced wildfire contributions on the winter MWT and ADD by up to 51%, while in the case of the Gibbon River site the fire-related decrease in the winter MWT and ADD was attenuated by 64%. The importance of weather conditions on the post-fire winter stream water temperatures was echoed in the study by Koontz et al. [28], which found that meteorological conditions accounted for a significant proportion of the post-fire change in seasonal and annual stream temperatures.

#### Summer season

The results from our multiple analyses indicated that the riparian burns within the Boulder Creek and Gibbon River watersheds corresponded to elevated MWTs and ADDs for studied downstream sites. Moreover, our RFR model-based results showed that the median fire-related increase in the summer MWT for the three subsequent years ranged between 0.39–0.56°C (*S*.*E*.< 0.17°C) and the median fire-related increase in the summer ADD ranged between 47–62 DDC (*S*.*E*.< 15.3 DDF) across the two stream sites. These findings are consistent with previous studies which have illustrated that the burning of riparian vegetation promoted the warming of summer water temperatures [e.g., [8, 9, 19, 21–23, 25, 59, 60]]. However, these changes appear modest compared to the fire-related increase in summer MWT and ADD reported in other wildfire studies [e.g., [8, 22]]. We ascribe this discrepancy to several factors. First, the two sites in this study have relatively large watershed areas. As such, any effect from fire-related increases in summer insolation on summer water temperatures might be attenuated by the volume of cool waters flowing through unburned upland areas. There is also substantial subsurface water contributions to the total summer flows at these stream sites, which could have a moderating influence on the rate of water heating (Table 1). Furthermore, the post-fire summer baseflow index (a measure of subsurface water contribution to total streamflow) for the Gibbon River site and the post-fire summer streamflow for the Boulder Creek site were significantly (*p*< 0.05) higher than pre-fire period (Table 2), which implies more summer water volumes that needed to be heated at these sites (Table 2). Additional factors such as differences in channel morphology, watershed and channel slope, and riparian and topographic shade could also have contributed to the smaller wildfire effect on summer MWTs and ADDs compared to other studies.

In contrast, we did not detect a warming effect from wildfire on the summer MWT and ADD for the Elk Creek stream site. This was despite the post-fire annual and seasonal air temperature and precipitation total showing no significant (*p*< 0.05) difference than pre-fire. We hypothesize that the absence of a significant post-fire increase in the summer water temperatures for the Elk Creek site was related to its distance (2.5 km) from the burned riparian area. Mahlum et al. [21] noted that large wildland fires did not affect the summer water temperature of sites that were 1.7 km downstream from them. They attributed this observation to the cooling effect of groundwater inflow and riparian vegetation downstream of the burn area. However, we note that the lack of a significant summer temperature increase for the Elk Creek stream site does not preclude localized warming of summer stream temperatures within the burn perimeter.

Our RFR model-based results indicated that weather accounted for 8–80% of the increase in the average post-fire summer MWT and ADD for studied stream sites. The greatest weather-related increase in the post-fire summer MWT and ADD was observed for the Elk Creek site. This highlights the importance of post-fire weather conditions in enhancing or muting the post-fire changes in the summer MWTs and ADDs for streams.

### Implications for coldwater fishes

Alterations to winter and summer thermal regimes, such as the winter decreases and summer increases observed at the Gibbon River and Boulder Creek sites, can alter fish phenological relationships in beneficial or detrimental ways. Salmonids are adapted to local climatic conditions; under those thermal regimes, development is timed so that life cycle transitions occur when physiology, behaviour, and morphology are sufficiently developed. The change in the rate of metabolic processes can alter timing rates of transition between life history stages in salmonids (e.g., egg hatching, fry emergence, smolt development, migration) [61–63]. Effects of shifted timing can have far reaching consequences beyond the life-stage directly impacted. For example, if timing of emergence from eggs is later or earlier by a few days, survival of juveniles may be influenced by biotic conditions such as available food or changes in abiotic conditions like water velocity, and even later life history stages could be delayed [62, 64]. Increased summer temperature, which occurs during a potentially bioenergetically stressful season for coldwater salmonids towards the upper end of thermal tolerance, could increase metabolic costs resulting in higher energy use and increased demand for prey [65]. For example, Beakes et al. [9] demonstrated how higher energetic costs from a 0.6 °C increase in post-fire water temperature over a summer, paired with a decrease in prey availability, resulted in lost energy reserves, individual mass loss, mortality, and emigration of rainbow trout (*Oncorhynchus mykiss*). In contrast, temperature increases at the lower end of thermal tolerance for salmonids can increase the growth rates in systems where growth is limited, but interactions can be complex. For example, changes in post-fire distribution and abundance of *O. mykiss* where stream temperatures warmed were the result of altered maturation and growth rates from interactions between higher temperatures, prey availability, competitive interactions, and bioenergetics [25].

Here we have focused on potential changes in stream water temperature from wildfire, but thermal regime change is only one of many transformations to stream habitat following wildfire [e.g., [8, 66, 67]]. Ultimately, the response of salmonid populations to post-fire thermal regimes will depend on the ecoregion, seasonal pattern, life history, behavioural adaption potential, and the concurrent changes in sediment regime, channel structure, water quality parameters, and other biota, as well as the connectivity and location of the site within the stream network [8, 66, 68]. To fully appreciate wildfire impacts on salmonid populations, a fuller accounting of these myriad processes is needed.

### Limitations of this study

Empirical studies are as good as the data and analytical methods used. This study utilized three separate statistical approaches of varying complexity to infer wildfire effects on winter and summer water temperatures in three WUS watersheds with extensive burns. Of the three analytical approaches, the bootstrap method was the simplest and most intuitive method of determining the significance of the deviation in the post-fire seasonal MWT and ADD relative to the pre-fire period and evaluating if this deviation corresponded to post-fire weather (air temperature, precipitation) patterns. However, it was also considered the weakest in that it did not allow for the parsing and quantifying of fire and weather influence on the post-fire change in the winter and summer stream temperatures.

In contrast, the air-stream water temperature regression analysis afforded characterization of fire effects on the winter and summer weekly air-stream water temperature relations. Moreover, our utilization of ARIMA regression models in this type of analysis was especially appropriate, as it accounted for autocorrelation effects when estimating the pre- and post-fire regression lines for weekly MWTs and ADDs. Nevertheless, the air-stream water temperature regression analysis of winter and summer weekly air-stream temperature relationship might not be considered the most robust of our approaches to study fire effects on the winter and summer MWT and ADD for two reasons. One was that the approach assumed that the weekly air-stream water temperature relationships were linear. Two, the approach did not include precipitation as a covariate when modeling weekly MWT and ADD, although advective heat input (either from runoff or groundwater) could be a major determinant of stream temperatures particularly during winter. This is perhaps why the coefficient of determinations (R^2^) for weekly winter MWT and ADD models were somewhat low at the Elk Creek and Boulder Creek sites.

We considered the random forest approach the most robust of the three methods because it (a) allowed non-linear modeling of winter and summer air-stream temperature relationships, (b) incorporated daily-to-seasonal weather variables as possible covariates for modeling daily MWTs and ADDs, and (c) enabled the parsing and quantifying of fire and weather contributions on the post-fire change in summer and winter MWT and ADD. Yet, LOOCV evaluations across our sites showed that this approach offered poor predictions of the weather-related change in MWT and ADD in seasons and sites where advective heat input either from runoff or baseflow had dominant control over the stream temperature. This implies that this approach might have limited applicability in estimating fire and weather contributions in these types of systems.

It is important to note that only a handful of studies [e.g., [8, 28]] have utilized statistical approaches, apart from the paired watershed comparison method, in evaluating the fire-related changes in stream temperatures. Our analysis, along with these studies, demonstrate the utility and efficacy of alternate statistical approaches in assessing stream temperature response to wildfires.

## Summary

Empirical assessments on the post-fire response of water temperatures across seasons, stream reaches, and watersheds help inform water resources managers that are concerned about wildfire impact on thermally sensitive fishes. In this study, we assessed the effect of wildfire burns in the Boulder Creek (Oregon), Elk Creek (Oregon), and Gibbon River (Wyoming) watersheds on the downstream water temperatures during the winter and summer seasons. To get results independent of the choice of approach, we employed three independent analytical method that use local water temperature data to evaluate the consequence of each burn on seasonal water temperatures. Our results indicate that for Boulder Creek and Gibbon River watersheds, wildfire burns were associated with the warming of summer water temperatures and the cooling of winter water temperatures in downstream sites. In contrast, the watershed burn at Elk Creek watershed did not correspond to substantial winter or summer water temperature changes, and the absence of any effect at this site was primarily related to the distance of the site from burned riparian areas. The use of analytical approaches that utilize local data allows the assessment of wildfire burn effects on stream water temperatures for other burned western US sites.

## Supporting information

S1 Table Summary of dataset attributes and sources used for this study. (DOCX)
