# Supplementary material for: Variable wildfire impacts on the seasonal water temperatures of western US streams: A retrospective study
Source: PLoS One. 2022 Jul 20;17(7):e0268452. doi: 10.1371/journal.pone.0268452 (PMC9299304; doi:10.1371/journal.pone.0268452)
Supplement: S1 Table — (DOCX) [file pone.0268452.s001.docx]

**Table S1. Summary of dataset attributes and sources used in this study.**

| Dataset | Description | Format (Standard) | Resolution | Period | Version Date | Source |
| --- | --- | --- | --- | --- | --- | --- |
| MTBS burned area boundaries | Burn Area | Spatial Vector (FGDC) | - | 1984-2018  (Annual) | 2020 | https://www.mtbs.gov/direct-download |
| MTBS Burn severity mosaic | Burn Severity | Spatial raster (FGDC) | 30x30 m | 1984-2018  (Annual) | 2020 | https://www.mtbs.gov/direct-download |
| GAGES II | Stream discharge (ft^3^/sec), water temperature (**°**C) | Time Series (WaterML) | - | 1979-2018 (Daily) | 2020 | https://doi.org/10.5066/F7P55KJN |
| GAGES II Geospatial Attributes | Watershed Boundaries | Spatial Vector (FGDC) | - | 2011 | 2017 | https://doi.or g/10.5066/F 7P55KJN |
| GRIDMET | Potential and Actual Evapotranspiration | Spatial raster (FGDC) | 4x4 km | 1979-2018 (Daily) | 2019 | https://climatologylab.org/datasets.html |
| NHDPlus V2 | Elevation (m), Slope (%), Area (m^2^) | Spatial Raster (FGDC) | 30x30 m | - | 2012 | https://www.epa.gov/waterdata/get-nhdplus-national-hydrography-dataset-plus-data |
| NLCD | Land Cover | Spatial Raster (FGDC) | 30x30 m | 2001 | 2011 | https://www.mrlc.gov/data/nlcd-2001-land-cover-conus |
| PRISM | Precipitation (mm), air temperature (C) | Spatial raster (FGDC) | 4x4 km | 1979-2018 (Daily) | 2020 | https://prism.oregonstate.edu/recent/ |
